# A Joint Approach of Morphological and UHPLC-HRMS Analyses to Throw Light on the Autochthonous ‘Verdole’ Chestnut for Nutraceutical Innovation of Its Waste

**DOI:** 10.3390/molecules27248924

**Published:** 2022-12-15

**Authors:** Elvira Ferrara, Maria Tommasina Pecoraro, Danilo Cice, Simona Piccolella, Marialuisa Formato, Assunta Esposito, Milena Petriccione, Severina Pacifico

**Affiliations:** 1 Dipartimento di Scienze e Tecnologie Ambientali Biologiche e Farmaceutiche, Università degli Studi della Campania “Luigi Vanvitelli” Via Vivaldi 43, 81100 Caserta, Italy; 2 CREA-Centro di Ricerca Olivicoltura, Frutticoltura e Agrumicoltura, Via Torrino 3, 81100 Caserta, Italy

**Keywords:** European chestnut, polyphenols, LC-HR MS/MS, waste management, circular economy, green extraction

## Abstract

Nowadays, chestnut by-products are gaining a lot of interest as a low-cost raw material, exploitable for developing added-value products. This is in line with suitable chestnut by-products’ management, aimed at reducing the environmental impact, thus improving the chestnut industry’s competitiveness and economic sustainability. In this context, with the aim of valorizing local cultivars of European chestnuts (*Castanea sativa* Mill.), our attention focused on the Verdole cultivar, which has been characterized by using the UPOV guidelines for its distinctness, homogeneity, and stability. After harvesting, Verdole chestnuts were properly dissected to collect the outer and inner shells, and episperm. Each chestnut part, previously crushed, shredded, and passed through diverse sieves, underwent ultrasound-assisted extraction. The extracts obtained were evaluated for their total phenolic, flavonoid, and tannin content. The antiradical capacity by DPPH and ABTS assays, and the Fe(III) reducing power, were also evaluated. Although all the samples showed dose-dependent antioxidant efficacy, plant matrix size strongly impacted on extraction efficiency. LC-HRMS-based metabolic profiling highlighted the occurrence of different polyphenol subclasses, whose quantitative ratio varied among the chestnut parts investigated. The outer shell was more chemically rich than inner shell and episperm, according to its pronounced antioxidant activity. The polyphenol diversity of Verdole by-products is a resource not intended for disposal, appliable in the nutraceutical sector, thus realizing a new scenario in processing chestnut waste.

## 1. Introduction

The sweet European chestnut (*Castanea sativa* Mill.), belonging to the Fagaceae family, is widely distributed in Europe with an invaluable cultural and historical heritage [1,2], so much so that its importance in the traditional economy has been referred to as “chestnut civilization” [3]. Thanks to its eco-pedological characteristics, it is appropriate for sustainable cultivation in hilly and mountain areas, providing the valorization of these marginal landscapes [4]. As the species plays an important role in the economic and environmental context of the food and wood industry, nowadays a new turning point leads to a revival in *C. sativa* cultivation [5]. Thus, the world production of chestnuts is growing, especially thanks to the high nutritional power of its fruit, suitable for consumption by non-celiac people sensitive to gluten and as an ingredient in health-related food products [6,7]. Around 2.3 million tons of fresh fruit were produced worldwide in 2020. China is the largest chestnut producer with 1.74 million tons, followed by Spain with 188,690 tons and Bolivia with 80,882 tons. Italy is the sixth largest producer in the world and the second in Europe with production of 49,750 tons [8]. 

This production is connected to a large number of chestnut shells which represent underutilized agricultural and forestry waste that can be exploited as a significant resource for the production of high-value natural active compounds [9,10]. In fact, as is known, the chestnut is covered with a shell that represents about 20% of its weight which is removed during the peeling process [11]. The removal of the shell, which is composed of the pericarp (outer shell), integument (inner shell), and episperm [12], produces multiple agro-residues that can be differently exploited for the extraction of high added-value bioactive compounds [10] and recyclable in different application fields (e.g., for fertilizing and feeding animals) [13,14,15]. In fact, low-molecular-weight phenols, such as gallic acid and protocatechuic acid [16,17], as well as hydrolysable and condensed tannins, and flavonoids have been found as constituents of chestnut shells [11,16,18,19,20,21], although an high variability in phenol content was found, ranging from 2.7 to 5.2% [20]. It is certain that the type of cultivar in the first place and then the soil and climatic conditions of cultivation, the harvest time, as well as the extraction conditions can massively influence the content of polyphenolic compounds [22,23]. Moreover, a polysaccharide portion can also be recovered from the chestnut shell. The abundance of these substances increases the value of the shells, and emphasizes, together with the presence of antioxidants, the potential benefits of designing high-added-value products with a circular economy approach [24].

In Campania, the first Italian region for chestnut groves with 13,800 hectares and an increase of 20–30% in areas destined for chestnut groves in recent years [25], chestnut cultivation is of considerable economic and social importance not only for fruit and wood production, but also for the conservation and hydrogeological landscape protection [26]. 

The recent certification of Marrone/Castagna di Serino PGI (Protected Geographical Indication) [27] has further innovated the chestnut production of the Campania Region with fruits of high-quality characteristics. In fact, Marrone/Castagna di Serino designates the fresh, peeled, dried in-shell, and whole dried shelled fruit of two varieties from European chestnut species: the ‘Montemarano’ variety, also referred to as ‘Santimango’, ‘Santomango’, ‘Marrone di Avellino’ or ‘Marrone avellinese’, and the ‘Verdola’ or ‘Verdole’ local variety. The Verdole variety, which also makes up 10% of the ‘Castagna di Montella’ PGI [28], and acts as a pollinator variety for its resistance to adverse environmental conditions and cryptogams, is the main chestnut cultivar grown in many low-altitude valleys near Serino (Avellino province). This makes this autochthonous variety a widely consumed product at a local level. Since the transformation of chestnuts leads to the production of an enormous quantity of waste and by-products, with a view to outlining new directions of use for local producers and the establishment of self-powered and sustainable supply chains, the study of phytochemistry has been carried out on the composition of the non-organic edible components of the Verdole cultivar. In addition to providing a series of guides and information for the distinctness, uniformity, and stability of Verdole chestnuts, traits of phenological and morphological characters have been evaluated.

Hence, in response to the current perspective of containment of agro-industrial waste, through the increase in the sustainability of production and the activation of an economic circularity of the company [29], the phytochemical study of the different components of the shell of the Verdole cultivar represents the first step for an effective enhancement of resources. For this purpose, samples of chestnuts of the Verdole variety have been collected and shelled. The shells, further separated into different components, have been subjected to differential pulverization procedures, and extracted by ultrasound-accelerated maceration using ethanol. The total content of condensed tannins, and the total content of flavonoids and phenols, as well as the anti-radical capacity, have been evaluated in each shell component. Furthermore, the relative metabolic profile has been recorded by means of a non-targeted Ultra-High Performance Liquid Chromatography–High Resolution Mass Spectrometry (UHPLC-HRMS).

## 2. Results and Discussion

### 2.1. Morphological Analysis

**Figure 1:**
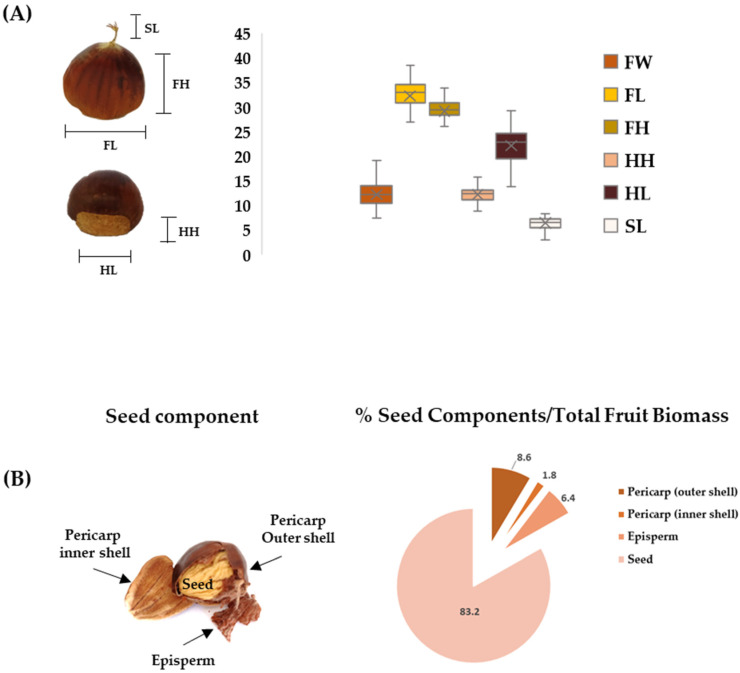
(**A**) Box and whiskers plot of the average values of morphological traits measures (*n* = 100) of chestnut fruit (FW: Fruit Weight; FL: Fruit Length; FH: Fruit Height; HH: Hilum Height; HL: Hilum Length; SL: Style Length). (**B**) Fruit components with relative %—Average Biomass (dry weight; *n* = 100 fruits).

*Castanea sativa* cv. Verdole was described using forty-seven morphological traits-descriptor for carrying out tests of the distinctness, homogeneity, and stability of chestnut established by the Union for the Protection of New Varieties of Plants (UPOV) guidelines (TG/124/4—UPOV 2017) [30]. For each mean value obtained, a state and numerical number were assigned using the UPOV as reported in Table S1 (Supplementary Materials). Verdole is a native variety of Campania Region and, as reported in Table 1 and Table S1, with medium tree vigor and upright habit. The bud of the current season is dense with long internodes and opposite leaf arrangements. Each shoot contains many female flowers and very long male flowers (catkins). The leaves are large and moderately asymmetrical with a slightly concave profile in cross-section The top side shows a medium intensity of green color while the bottom side appears as light green. The shape of a leaf is narrow and elliptical with a sharp apex and a corded base. The burrs have an obloid shape with a low density of thorns and contain two or three fruits. It produces a red-brown colored nut with a single embryonic seed and medium penetration of the epidermis. With regard to pomological traits, according to Figure 1A the box indicates the 25th and 75th percentiles and the whiskers represent the maximum and minimum values of fruits. The fruits’ average weight is 12.2 g ± 2.3 with the fruits’ width and height of 32.6 ± 2.6 mm and 29.5 ± 1.6 mm, respectively. The hilum height and length are 12.3 ± 1.3 mm and 22.3 ± 3.1 mm, respectively. The style and the flower remaining part show a height of 6.3 ± 1.2 mm, while the seeds evidence the adherence to the core and the yellowish color of the pulp. This variety has a medium-term leaf budding and the early male flowering-period and the average female flowering ripening-period occurs towards the end of September and can be considered medium. Fruit is suitable for fresh consumption due to its organoleptic and sensorial characteristics. Many of these characteristics are stable and the evaluation of a single year in a single site is satisfactory, as already demonstrated on observations carried out on thirty-eight traditional chestnut cultivars from a contemporary collection in the northwest of the Iberian peninsula [31]. On the contrary, phenological parameters (time of leaf bud burst, time of beginning of male and female flowering) and one related to fruit size (size of fruit hilum) need to be evaluated under contrasting site conditions for a minimum number of years as it has been demonstrated that they can vary in relation to environmental conditions [31,32]. 

Herein, for a greater and more in-depth detail of the characteristics of the fruit, the individual components of the integument and the seed were accurately dissected. Verdole cv. chestnuts were shelled, and shells acquired were dissected in its outer and inner part, as well as episperm (Figure 1B). It was found that, in addition to the edible seed which was 83.2%, the outer shell accounted for 8.6%, the inner shell 1.8%, and the episperm for 6.4%.

### 2.2. Extraction of Chestnut Fruits

The extraction method mostly affects the recovery of bioactive compounds from plant matrices [33]. Several parameters, such as solid–liquid ratio, particle sizes, solvent polarity, temperature, extraction time, and pH impacted on the extraction efficiency [18,34,35,36]. The literature data highlighted that different extraction methods were applied to chestnut shells [18]. As a result, significant differences in the qualitative and quantitative phenolic patterns of chestnuts shells were found. The large amount of literature data clashed with the scarce information relating to the cultivar, the geographical origin, and the collection site of the chestnuts whose shell polyphenolic component was studied. The chestnut shell represents a processing waste for the processing industries that must be disposed of, where it could generate new economic and social profit [37]. Table 2 shows the literature search of different papers that used chestnut shells as the starting matrix.

Starting from recovered Verdole cv. chestnut shells, the impact of particle size on extraction efficiency was preliminarily investigated, and the use of sieves was designed to prepare sequentially four outer shell matrices (P_os_1–P_os_4). The latter were obtained in the relative percentage of 47.5% (P_os_1), 21.6% (P_os_2), 7.9% (P_os_3), and 4.3% (P_os_4) with respect to the previously shredded outer-shell matrix. Thus, each component underwent extraction using ultrasound-assisted maceration and ethanol as an extractant. 

**Figure 2:**
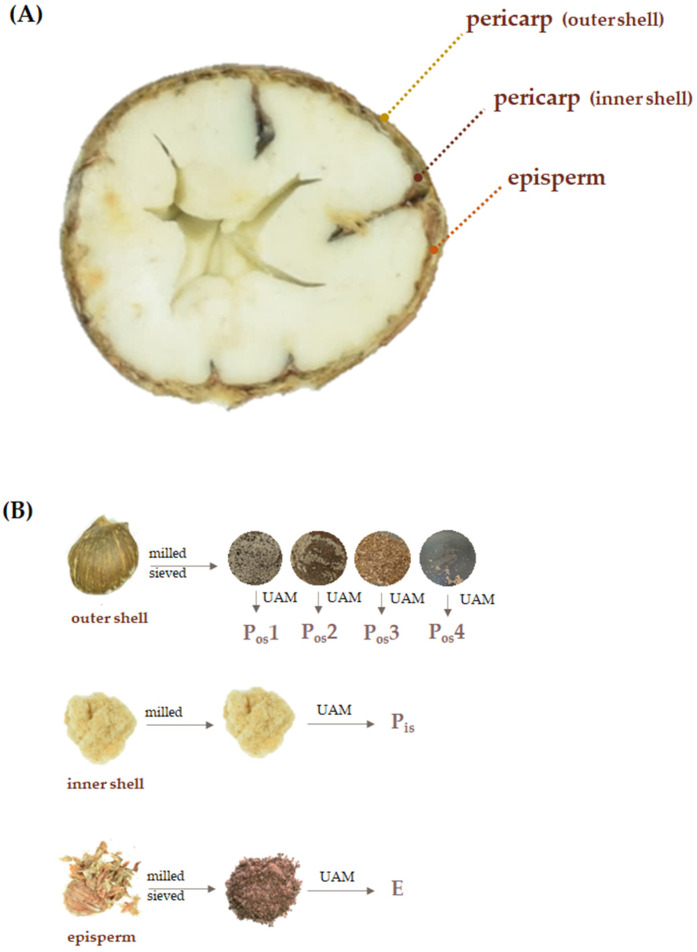
(**A**) Cross dissection of chestnut fruit; (**B**) Separation of the outer shell, inner shell, and episperm from the seed. Outer shell was sieved and different granulometric fractions were obtained. All the fractions underwent UAM (Ultrasound Assisted Maceration) using ethanol.

The reduction of the matrix to fine powders was in line with the relative increase in the specific surface area and appeared to be a guarantee for effective diffusion of the solvent within the matrix and greater mass transfer. Indeed, considering the percentage data from the shredded outer shell matrix, while observing that the extraction efficiency for the same weight of the differently sieved initial matrix increased with the decrease in the particle size, passing from 1.7 (P_os_1) to 5.7% (P_os_4), it is assumed that the processing of outer shell with particles larger than 1 mm, leads to a good recovery of bioactive compounds (Figure 2B).

### 2.3. Shell Components Differ in Phenols and Flavonoids, and Otherwise Act as Antioxidants

**Figure 3:**
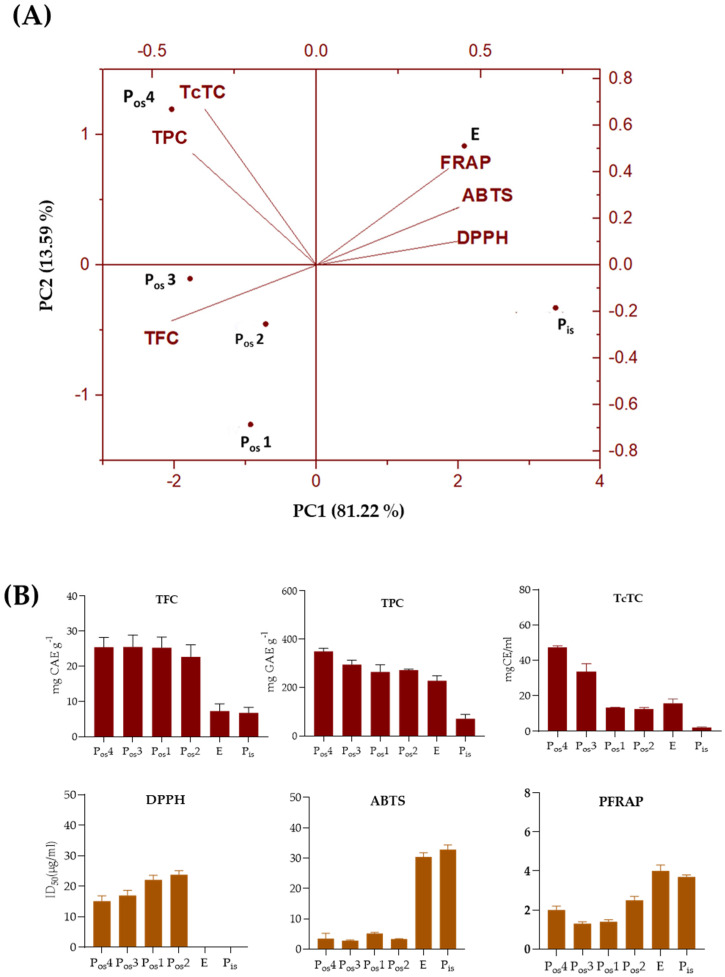
(**A**) PCA of antioxidant assays (DPPH, ABTS, and ferricyanide FRAP) and total content in phenols (TPC), flavonoids (TFC), and condensed tannins (TcTC) of the sampled shell components. Data processing was performed using OriginPro 2015 software. (**B**) Data of antioxidant assays, phenols, flavonoids, and condensed tannins of the analyzed shell components are ordered according to the gradient of the first axis (performed by GraphPad Prism 8.4.2.).

The different components of the chestnut shell of the cultivar Verdole were investigated in terms of total phenols (TPC), total flavonoids (TFC), and total condensed tannins (TcTC) (Figure 3). The anti-radical and Fe(III) reducing efficacy was also evaluated. The Principal Component Analysis of the data matrix performed with the values of the parameters showed, above all, a significant difference between the type of matrices examined. Episperm was at the positive end of the first axis, which reaches a dissimilarity value of 81.22%. On the contrary, the outer shell samples were pooled in the negative score. In particular, the P_os_4 extract showed a positive correlation with TPC and TcTC labels, whereas P_os_3 and P_os_2 appeared to be positively correlated with flavonoid content (TFC). 

The data were compared with those already present in the literature for this type of processing waste. Condensed tannins, ellagitannins, phenolic acids, and flavonoids are reported as the main constituents [42], to which health-promoting effects against oxidative stress-related disorders are attributed [54]. In this context, it was observed that the alcoholic extracts from the differently sieved outer shell, were able to scavenge stable radicals, such as the DPPH^•^ and ABTS^•+^, whereas extracts from the inner shell and episperm were inefficacious to reduce DPPH radical and exerted an ABTS^•+^ scavenging capability six-fold lower than that from P_os_1. This latter scavenged both the radical species less than extracts obtained using sieves with smaller meshes. Data from the ferricyanide FRAP assay highlighted that all the extracts contained compounds able to transfer a single electron to ferric ions.

The polyphenol content (TPC), evaluated by the Folin–Ciocalteu test, ranged between 393.42 mg GAE/g DW in the outer shell and 70.19 mg GAE/g DW in the inner shell, while the TPC of the chestnut episperm was 227.43 mg GAE/g DW (Figure 3B). This finding was in line with previous findings related to the non-uniformity of the distribution of phenolic compounds in plants. Typically, the outer layers contained higher levels of phenols than the inner ones [55]. 

Particle size fractionation was effective in producing four powdered outer shell materials with an average phenol and flavonoid content of 294.54 mg GAE/g DW and 24.71 mg EC/g DW, respectively. The results acquired were comparable to the polyphenol content in the C. sativa shell extract obtained using ultrasound-assisted extraction at 70 °C for 40 min (393.1 mg GAE/g DW) [41], subcritical water extraction (315.21 mg GAE/g DW to 496.80 mg GAE/g DW) [42], and conventional extraction methodologies [14,40,56]. Higher TPCs were obtained in the MeOH and EtOH shell extracts of Italian chestnut cultivars with values of 870.81 mg GAE per gram of extract and 547.85 mg GAE per gram of extract, respectively [39]. Lower TPC values were obtained from the outer shell when treated with aqueous alkaline solvents [16,46] or with organic solvents of different polarity [41,46,57]. 

The chestnut episperm showed a higher TPC than that determined by Squillaci et al. [16] when the inner part of the shell was removed from the “Brulage” peeling process, while a higher TPC was detected in the ethanol extraction by Silva et al. [50]. The flavonoid content was higher in the outer shell and episperm than in the inner shell (Figure 3B). Previous studies reported a TFC of 6.5 mg CE/g DW and 14.4 mg CE/g DW [48], and of 7.94 mg CE/g DW and 40.98 mg CE/g DW [16] for the episperm and outer shell, respectively. A lower TFC value was reported by Hong et al. [57] for chestnut shell extract including internal and external shells. Pinto et al. [42] found a TFC between 25.50 mg of CE/g DW and 68.56 mg of CE/g DW in the ethanolic and aqueous extract of chestnut shells, respectively. On the contrary, Vella et al. [49] determined a significantly lower TFC for the outer shell compared to the inner shell even after employing longer extraction periods and higher temperatures. 

### 2.4. UHPLC-HR-MS/MS Analysis

According to previous findings, hexuronic acid and its derivatives were found as the main constituents of inner shell and episperm, while diverse hydrolysable and condensed tannins were detected as constituents of the investigated chestnut materials, together with flavonoid compounds [38]. This latter appeared the most abundant class in all the outer shell extracts. This could be due to the use of ethanol as an extractant.

**Figure 4:**
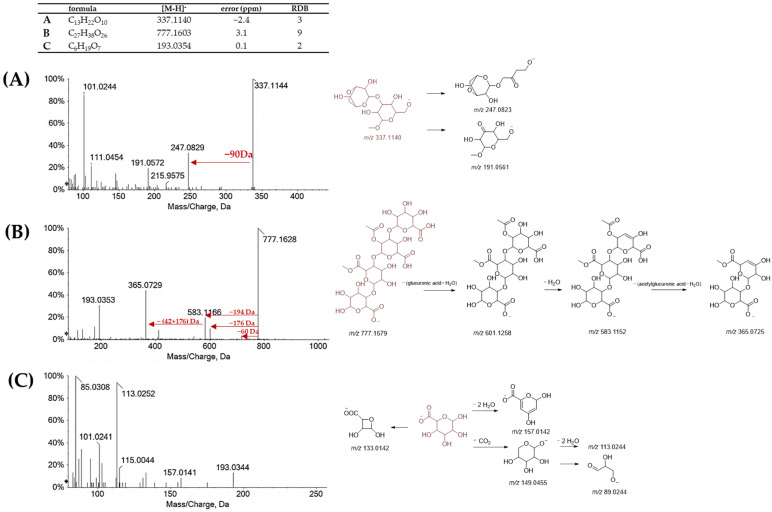
ToF-MS/MS spectrum of saccharidic compounds detected in inner shell and episperm extracts (**A**) compound A; (**B**) compound B; (**C**) compound C. Next to each spectrum, the proposed fragmentation pattern is represented. ToF-MS values, as well as RDB (ring and double bond value) and error (ppm), are tabulated.

The high content of hexuronic acid, mainly at the inner shell level, was in line with previous observations relative to the content of galacturonic acid [38]. Furthermore, investigating the monosaccharide content of the shell of four chestnut varieties (Bouche de Betizac, Marigoule, Goujounac, and Bournette), it was found that xylose and galacturonic acid were the most representative. Herein, the evidence of the compound was through the detection of the deprotonated molecular ion at *m/z* 193.0354 (compound C; Figure 4), which underwent decarboxylation to achieve the ion at *m/z* 149.0436, and further dehydrated twice for giving the ion at *m*/*z* 113.0251. A galacturonyl-based compound was also compound B, with relative [M-H]^−^ ion at *m*/*z* 777.1603. This latter fragmented in the ToF-MS/MS experiment providing the main ions at *m/z* 583.1166, and 365.0729, according to sequential neutral losses of hexuronate, and acetyldehydrohexuronate. ToF-MS/MS data of compound A was tentatively identified as methyl 3-O-(3,6-anhydrohexopyranosyl) hexopyranoside. 

Galacturonic acid is a very versatile compound used extensively in the food industry. In fact, it acts as an acidifying agent, is a good sequestrating agent, and was proposed as an ingredient in cosmetics and pharmaceuticals. Recently, PCS-2A, a polysaccharide with 62.8% galacturonic acid, was isolated from the chestnut shell, and this high rate of galacturonic acid was due to its antioxidant capacity, and hepatoprotective effects [58]. Dietary pectin represents the main source of galacturonic acid [59], and its benefits appeared to ameliorate the intestinal mucosal permeability and inflammation of functional dyspepsia [60]. Considering the many uses in which galacturonic acid is involved, which is also a precursor in the synthesis of vitamin C, the recovery of galacturonic acid is of great interest [61] and the chestnut inner shell is a precious resource. In fact, among the constituents detected in this waste part, it represented more than 80%.

The other shell parts, as previously described mostly accounted for tannins and flavonoids, beyond dihydroxybenzoic acid (**7**) with the [M-H]^−^ ion at *m*/*z* 153.0200, according to the molecular formula C_7_H_6_O_4_, and the lignan hexosides, **17** and **20**. These compounds showed the deprotonated molecular ion at *m/z* 521.2020 and 521.2046, respectively, and ToF-MS/MS ions (at *m*/*z* 359.15, and 344.13), in accordance with the loss of dehydrated hexose. Isolariciresinol and lariciresinol glycosides were previously detected in sweet chestnut flour [62]. 

In Table 3, compounds, whose structure is based on gallic acid or hexahydroxydiphenic acid, are reported. In this case, the diversity in hydrolysable tannins, which are esters of gallic acid or hexahydroxydiphenic acid, is simplified in the description of the molecules of this class, mostly present in the extracts [63]. 

**Figure 5:**
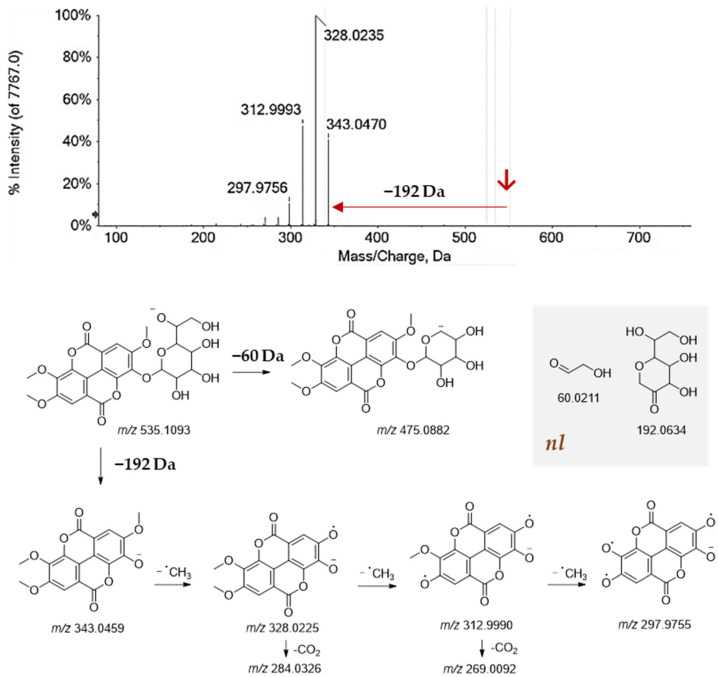
ToF-MS/MS spectrum of trimethylellagic heptuloside (**47**), and fragmentation pattern proposed. nl = neutral loss. The theoretical mass is below each structure.

The ToF-MS and ToF-MS/MS data of compound **1** were in accordance with the hexahydroxydiphenoyl hexose. In fact, the [M-H]^−^ ion at *m*/*z* 481.0634, underwent dehydrated hexose loss to achieve the ion at *m*/*z* 300.9994, which consisted of hexahydroxydiphenic acid (HHDP). Compound **4** was tentatively a galloyl derivative of compound **1**, whereas compound **9** was diHHDP-hexose, to such an extent that the dissociation of its deprotonated molecular ion at *m*/*z* 783.0709 provided the fragment ion at *m/z* 481.0627 in line with HHDP-hexose, and the ions at *m*/*z* 300.9984 (HHDP), and the decarboxylated HHDP ion at *m*/*z* 275.0191. Furthermore, compound **10** was also strictly related to the previous ones, being constituted by another HHDP unit. In this context, castalagin and its isomer vescalagin were previously identified as chestnut constituents [53]. These compounds were the most abundant ellagitannins in freshly felled chestnut heartwood [64]. The loss of the HHDP residue was further common to compound **31**, whose deprotonated molecular ion, after losing 302 Da, provided the ion at *m*/*z* 447.0579, which in turn lost 146.06 Da, in line with a deoxyhexose, to achieve the hexahydroxydiphenate. Compound **14** was likely castacrenin. In fact, its deprotonated molecular ion gave the ion at *m/z* 493.0076 following the cleavage of the hydroxytetrahydrofuranyl ethanediol moiety and the loss of 120 Da. Ellagic acid (**26**) was the most abundant compound, whereas its methyl-(**41**), dimethyl (**44** and **46**), and trimethyl derivatives (**47** and **51**) were also detected. The deprotonated ion of compound **47** by losing the heptosyl unit (−192 Da) gave the deprotonated trimethylellagic acid which underwent three sequential losses of methyl radicals (Figure 5). Heptulose is a seven-carbon atoms monosaccharide, rarely found in nature [65], and herein identified for the first time as a glyconic moiety of trimethylellagic acid. This latter compound was recently investigated for its ability to exert antiproliferative activity towards different cell lines and to inhibit the growth of SW620 tumor xenografts in vivo by inducing apoptosis and anti-angiogenesis [66]. The great part of the most polar compounds in investigated chestnut shell extracts were other galloyl-based compounds. Indeed, gallic acid (**3**) and its hexoside (**2**), as well as a digalloylhexose (**15**), and two isomers of trigalloylhexose (**18** and **19**) were identified. Neutral losses of 170 and 152 Da, together with the detection of the fragment ions at *m*/*z* 169.01, and 125.02 mostly allowed the identification of these compounds. The ToF-MS/MS spectrum of the trigalloylhexose **18**, in particular, showed the deprotonated molecular ion [M-H]^−^ at *m/z* 635.0890, which was beyond typical neutral losses of 152 Da and 170 Da, underwent the loss of 212 Da suggesting the location of the galloyl residues in positions 1, 3, and 5 of the saccharide portion. In fact, the 212 Da loss could be the result of a cross-link sugar cleavage (Figure 5). Compound **5** was tentatively identified as a trihydroxybenzyl alcohol hexoside, likely crenatin, previously identified as one of the main compounds of the Italian PGI chestnut ‘Marrone di Roccadaspide’ [67].

Proanthocyanidins, and their monomers catechin (**12**), gallocatechin (**8**), and (epi)catechin gallate (**22**), were tentatively identified (Table 4). Compound **6** was likely a B-type proanthocyanidin, formed by (epi)gallocatechin monomers. The deprotonated molecular ion [M-H]^−^ at *m/z* 609.12 agreed with the molecular formula C_30_H_26_O_14_. The neutral loss of 126 Da, consisting of the A ring loss of the monomer, supplied the ion at *m*/*z* 483 through an HRF (heterocyclic ring fission) mechanism, whereas the ion at *m*/*z* 305.0665, corresponding to the monomer unit, was formed by the mechanism of fission QM (quinone methide) with cleavage of the interflavanic bond. The loss of the B ring following a Diels Alder retro reaction confirmed the presence of the pyrogallol unit. The other three compounds (**D**–**F**) were found to exhibit the deprotonated molecular ion at *m*/*z* 609.125. These compounds were detected only in the episperm extract. Compound **D** showed a ToF-MS/MS spectrum superimposable to that of compound **6**, while compounds **E** and **F** appeared to be catechin or epicatechin derivatives. 

Furthermore, compound **13** was a B-type procyanidin; it shared a fragmentation pattern the same as compound **6**, showing catechin as the monomeric unit. Compounds **21** and **32** were galloyl derivatives of the B-type procyanidin. 

**Figure 6:**
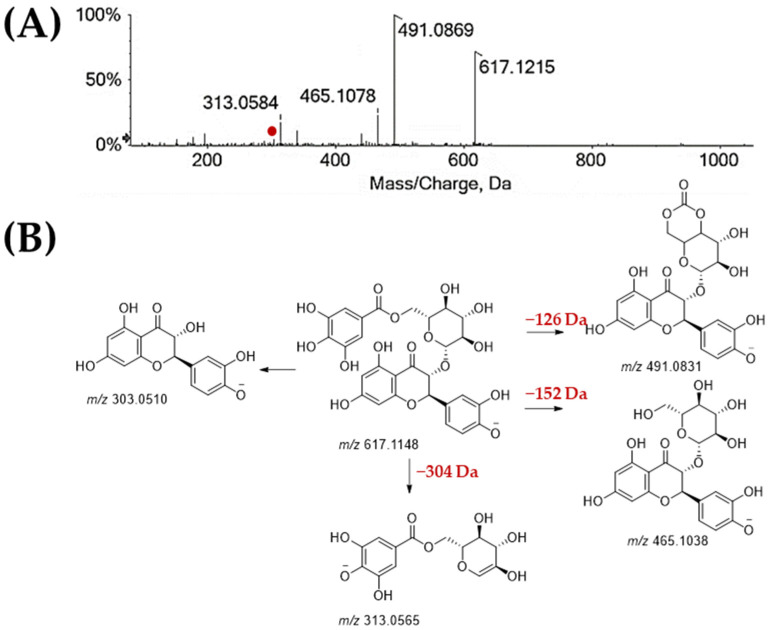
(**A**) ToF-MS/MS spectrum of the dihydroquercetin galloylhexoside (**25**), and (**B**) fragmentation pattern proposed. Theoretical mass is below each structure.

Flavonol, flavandiol, and flavone glycosides were also found, some of which were never reported before from chestnut shells. Quercetin, isorhamnetin, and myricetin were the main flavonol aglycones (Table 5). Myricetin hexoside (**24**) and myricetin deoxyhexoside (**26**), were dihydroflavonols together, such as dihydrokaempferol glucoside (**23**) with deprotonated molecular ion at *m*/*z* 449.1097, which underwent neutral loss of 162.05 Da (to give the ion at *m*/*z* 287.0557, consisted in dihydrokaempferol) and water to achieve the base peak at *m*/*z* 269.0444. The galloyl derivative of isoquercetrin (**28**) was further tentatively identified. It showed the deprotonated molecular ion at *m*/*z* 615.1050, which underwent 152 Da neutral loss to give deprotonated isoquercetrin at *m*/*z* 463.0907. This latter was also identified (**30**), together with deoxyhexosyl (**35**) glycoconjugates. Dihydroquercetin (**34**) with [M-H]^−^ ion at *m/z* 303.0514 was also identified, as well as its galloylhexose compound (Figure 6), likely taxillusin (**25**). In fact, when the deprotonated molecular ion dissociated the ions at *m*/*z* 491.0869 and 465.1078, through neutral losses of 126 and 152 Da, respectively, the ion at *m*/*z* 313.0584 was tentatively the galloyl hexose-H_2_O residue, while that at *m*/*z* 303.0520 corresponded to dihydroquercetin. Several studies stated that dihydroquercetin exerts an Nrf2/HO-1-mediated anti-inflammatory effect and shows also hepatoprotective, cardioprotective, and neuroprotective activity [68]. 

Isorhamnetin glycosides, identified based on characteristic sugar losses of 308 (rutinose—H_2_O; **36**), 162 (hexose—H_2_O; **42**), 146 (deoxyhexose—H_2_O; **37**), and 132 Da (pentose—H_2_O; **41**), were the most representative among the identified methoxyflavonoids. In fact, compounds **33**, **39**, and **43**–**49** were in accordance with methoxylated flavonoids, whose methylation degrees were suggested based on the number of methyl radicals lost. In particular, compounds **33**, **39**, and **43** were both methoxylated and glycosylated, whereas the other compounds were detected as aglycones. Furthermore, phloretin glucoside (**29**) and naringenin gallate (**38**) were also tentatively identified.

**Figure 7:**
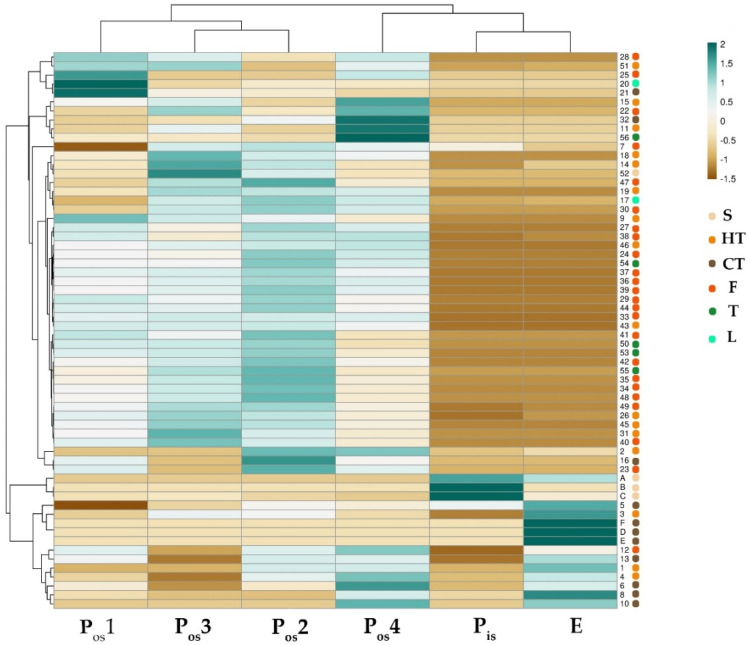
Heatmap of the compounds tentatively identified in the alcoholic extracts from Verdole cv. shell. S = sugars; HT = Hydrolysable Tannins; CT = Condensed Tannins; F = Flavonoids; T = Triterpenes; L = Lignans. Annotations on top of the heatmap show clustering of the investigated samples. In the ClustVis hierarchical clustering tool, both rows and columns are clustered using correlation distance and average linkage.

Although flavonoid occurrence was recently unraveled through an untargeted mass spectrometric analysis [43], proanthocyanidins were found as the most abundant polyphenol class both in terms of numbers and areas in E and P_os_4, respectively, as displayed in Figure 7. Herein, probably due to the extraction strategy applied, flavonols were the most abundantly detected compounds, mainly in the outer shell (P_os_4,P_os_3,P_os_2). This finding also suggests, in this case, that a fine treatment of the outer shell sample is desirable to optimize an exhaustive recovery of these compounds, which can exert a massive protective action that goes well beyond the much-claimed antioxidant activity. Long-term oral administration of myricetin demonstrated glycoregulatory activity [69], and isorhamnetin was found to reduce diabetes-related disorders by decreasing glucose levels, improving oxidative status, relieving inflammation, and modulating lipid metabolism and adipocyte differentiation [70]. Furthermore, quercetin has been investigated for treating metabolic diseases, including diabetes, hyperlipidemia, and nonalcoholic fatty liver disease [71]. These are just some of the beneficial effects enhanced by in vitro and in vivo studies on these compounds. The availability of a high quantity of waste shells in a local cultivar suggests their full recovery and the feeding of different supply chain paths with a big impact on the territory. Analogously, the presence of triterpenes (**52–56**) (Table 6), although negligible with respect to other detected compounds, can support the isolation of substances with a peculiar chemopreventive action. In fact, ursane-type triterpenoid saponins from the sweet chestnut heartwood were observed to be cytotoxic to prostate cancer cells and breast cancer cells [72].

Relative quantitation, considering each compound area in the investigated extracts, highlights extraction is forced by the intrinsic nature of the chestnut shell part, and, for the outer shell component, by the size of the plant matrix, obtained using sieves with gradually smaller dimensions. The particle size strongly influenced the extraction efficiency and the relative abundance of the compounds. In fact, the metabolic profile based on UHPLC-HRMS showed the presence of different subclasses of polyphenols, whose quantitative ratio varied between the parts of the chestnut studied. 

## 3. Materials and Methods

### 3.1. Chestnut Samples

*C. sativa* Mill. cv. Verdole, collected in a commercial orchard located at Serino, Avellino, Italy (40°80′68.1″ N; 14°89′62.9″ E), was analyzed. The orchard includes 100 trees per hectare. Morphological and phenological traits of ‘Verdole’ chestnut were detected on 50 trees using a guideline for the tests of distinctness, uniformity, and stability to chestnuts (*C. sativa*) TG/124/4 in agreement with UPOV (2017) [30]. Chestnut fruits were harvested on 20 October 2021. Fruits were shipped to the laboratory, checked for physical and biotic characteristics, and immediately hand-peeled to obtain the pericarp and episperm separately. The pericarp was in turn dissected into the outer and inner shells. All the samples were weighed and dried in a thermo-ventilated oven at 40 °C for 72 h and after milled using a mill (Sorvall DuPont Omni Mixer, United States). From the outer shells, using a vibrating sieving apparatus (Retsch, Haan, Germany) (53–250 µm; 250 µm–500 µm; 500 µm–1500 and >1 mm), four fractions with particles of different sizes were obtained. Each fraction was weighed and stored for further analysis.

### 3.2. Extraction of Verdole cv. Chestnut 

Chestnut inner and outer shell samples were extracted using ethanol 96% as the extracting solvent. The sample/solvent ratio was 1:10 (3 g sample in 30 mL of solvent). Three extractions cycles were carried out by maceration interspersed with 24-h periods at 4 °C under continuous stirring. At each extraction cycle, the ethanol fraction was filtered, dried by rotavapor (Heidolph Hei-VAP Advantage, Schwabach, Germany) and then weighed to evaluate the extraction percentage yield. The samples were stored at 4 °C until further analyses.

### 3.3. Determination of DPPH and ABTS Radical Scavenging Capacity

The alcoholic extracts were tested towards 2.2-diphenyl-1-picrylhydrazyl (DPPH) and 2.2′-azinobis-(3-ethylbenzothiazolin-6-sulphonic acid (ABTS) for the outer pericarp (1.5, 3.125, 6.25, 12.5, 25, 50, 100 and 200.0 μg/mL final concentration levels in dimethyl sulfoxide) and for the inner pericarp and episperm (0.375, 0.75, 1.5, 3.125, 6.25, 12.5 and 25.0 μg/mL final concentration levels in water). The DPPH solution was prepared as reported by Pacifico et al. [73]. After 15 min, the absorbance at 515 nm was read using a Victor3 spectrophotometer (Perkin Elmer/Wallac; Waltham, MA, USA). ABTS was prepared as previously reported [74]. The solution was then diluted with Phosphate Buffered Saline (PBS; Gibco^®^, Grand Island, NY, USA; pH 7.4) to achieve an absorbance of about 0.70 at 734 nm. The extracts were incubated in ABTS solution for 6 min and the absorbance at 734 was detected. Trolox (4, 8, 16, 32 µM) served as the positive control in both antiradical assays. Three replicate measurements were taken for each sample (three for each concentration). The results were expressed as the effective concentration of sample required to scavenge DPPH and ABTS radicals by 50% (ID_50_ value).

### 3.4. Determination of Fe (III) Reducing Power

To estimate the reducing power of the investigated alcoholic [75] the chestnut extracts (0.375, 0.75, 1.5, 3.125, 6.25, 12.5, 25.0 and 50.0 μg/mL, final) were dissolved in DMSO and in a NaH_2_PO_4_/Na_2_HPO_4_ buffer (0.2 M. pH 6.0. 2.5 mL). Samples were incubated at 50 °C for 30 min. Then, an aqueous trichloroacetic acid solution (100.0 mg/mL) was added. The tubes were placed on a shaker plate; after 10 min, 2.5 mL of the reaction mixture was taken and added to 2.5 mL of MilliQ water and 0.5 mL of ferric chloride (1.0 mg/mL). The absorbance of samples was monitored at 720 nm. The results were expressed as the effective concentration of the sample required to reduce Fe(III) by 50% (ID_50_ value). 

### 3.5. Determination of Total Phenols and Total Flavonoids

The Folin–Ciocalteu method was used to determine the total phenols in several extracts [76]. Each extract (1 and 0.5 mg) was added with an assay solution containing 2.25 mL of Na_2_CO_3_ (7.5% *w*/*v*) and 0.250 mL of the Folin–Ciocalteu reagent (FCR). Samples were stirred for 3 h at room temperature. and the absorbance was read at 765 nm by a 96-well microplate using a UV-1700 spectrophotometer (Shimadzu, Salerno, Italy). Results were expressed as milligram gallic acid equivalents (GAEs) per 100 g of dry material. Total flavonoid content (TFC) was performed using the aluminum chloride colorimetric assay [77]. Sodium nitrite (5%, *w*/*v*; 0.3 mL) was first added to the samples (1 and 2 mg), previously solubilized into 5 mL of distillate water, and then AlCl_3_ solution (10%, *w*/*v*; 0.6 mL). After 6 min, NaOH aqueous solution (1.0 M, 2.0 mL) was added, and the mixture was further diluted to 10 mL with distillate water. The absorbance was read at 510 nm against the blank (water) using a Synergy spectrophotometer (Biotek, Winooski, VT, USA). The results were expressed as milligrams of catechin equivalents per 100 g of DW. 

### 3.6. Determination of Condensed Tannins

Condensed tannins from the different chestnut samples (outer pericarp, inner pericarp, and episperm) were determined in agreement with Porter [78]. Tannin extract was diluted in acetone 70% (*v*/*v*) then, butanol-HCl reagent (butanol-HCl 95:5 *v*/*v*) and a ferric reagent (2% ferric ammonium sulfate in HCl 2N) was added. The mixture assay was vortexed and put in a heating block at 100 °C for 60 min. The absorbance of detection was 550 nm. The results were expressed as µg/mL using catechin as standard.

### 3.7. UHPLC-ESI-QqTOF-MS/MS Analysis

A Shimadzu NEXERA UHPLC (Shimadzu; Tokyo, Japan) system was used with a Luna^®^ Omega C18 column. A linear gradient was applied for this aim and water (A) and acetonitrile (B), both with 0.1% formic acid. Gradient conditions were as follows: 0–3 min. linear from 2 to 7% B; 3–7 min, linear from 2 to 10% B; 7–15 min, from 10 to 30% B; 15–20 min, from 30% to 45% of B; pumping 20–23 from 45% to 55%. The initial conditions were then restored at 23.01 min and allowed to re-equilibrate for 2 min. The flow rate was of 0.5 mL min^−1^ and the injection volume of 2.0 μL. MS analysis was carried out using the AB SCIEX TripleTOF^®^ 4600 (AB Sciex. Concord, ON, Canada), equipped with a DuoSpray^TM^ ion source, operating in ESI negative mode. The APCI probe was used for automated mass calibration using the Calibrant Delivery System (CDS). The CDS injected a calibration solution matching the polarity of ionization and calibrated the mass axis of the TripleTOF^®^ system in all scan functions used (MS and/or MS/MS). The QqTOF HRMS method, which combines TOF-MS and MS/MS with Information Dependent Acquisition (IDA), consisted of a full scan TOF survey (accumulation time 250 ms, 100–1500 Da) and a maximum number of eight IDA MS/MS scans. The MS parameters were as follows: curtain gas 35 psi; nebulizer gas (GS 1) 60 psi; heated gas (GS 2) 60 psi; ion spray voltage 4.5 kV; interface heater temperature 500 °C; declustering potential −70 V. Collision Energy (CE) applied was −35 V with a CE spread of 15 V. The instrument was controlled by Analyst^®^ TF 1.7 software, while data processing was carried out using PeakView^®^ software version 2.2.

### 3.8. Statistical Analysis

All data were expressed as mean ± standard deviation (SD). The test was carried out by performing three replicate measurements for three samples (*n* = 3) of the extract (in total 3 × 3 measurements). Statistical analysis was performed using Graphpad Prism 8 software (Graphpad Software. La Jolla, CA, USA). Differences between average values were assessed based on the Tukey test at a confidence level of 95 % (*p* < 0.05). A multivariate analysis approach by ClustVis (https://biit.cs.ut.ee/clustvis/) was adopted to explore and clarify the quali-quantitative compositive data compounds in the chestnut waste. PCA analysis was processed using OriginPro 2015 software. 

## 4. Conclusions

The Verdole cultivar, highly appreciated by the local communities, is an accessory cultivar from the Campania Region. Herein, a careful investigation of its agronomic traits and of bioactive compounds in the non-edible fruit parts, aimed at their recovery, was carried out. Ultrasound-assisted maceration in ethanol allowed the preparation of extracts enriched in saccharides or flavonoids, providing new insights into commonly treated matrices for the recovery of the tannic component. Since the waste of the Verdole chestnut cultivar, as well as other more or less valuable cultivars, is considerable, the depletion of the matrices through sustainable extractions could otherwise benefit the nutraceutical sector. The comparison of the data obtained with those in the recent literature underlines the need to perform an initial agronomic evaluation of the different chestnut cultivars to define the intraparietal and interspecific variability of bioactive compounds, whose recovery, with a view to territorial sustainability, could be the starting point for new chains of value.

## Figures and Tables

**Table 1:** *Castanea sativa* cv. Verdole morphological traits according to UPOV guideline.

*VERDOLE* DESCRIPTOR ATTRIBUTED		
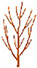	Tree: vigor Tree: growth habit	*Medium* *Upright*
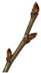	Current season’s shoot: thickness Current season’s shoot: length of internodes Current season’s shoot: arrangement of leaves Current season’s shoot: color of upper side stem Current season’s shoot: density of lenticels Shoot: number of female flowers	*Thick* *Long* *Opposite* *Yellow brown* *Dense* *Many*
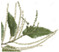	Male flower: length of filament Catkin: length	*Very long* *Long*
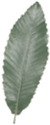	Young leaf: bronze coloration Leaf: size Leaf: profile in cross-section Leaf: symmetry Leaf: length/width ratio Leaf: attitude in relation to shoot Leaf blade: green color intensity of upper side Leaf: color of lower side Leaf: shape Leaf: shape of apex Leaf: the shape of base Leaf: shape of margin Leaf: symmetry of base Leaf: color of petiole Leaf: ratio length of leaf blade/length of petiole	*Absent* *Large* *Slightly concave* *Moderately asymmetric* *High* *Upwards* *Medium* *Light green* *Narrow elliptic* *Broad acuminate* *Cordate* *Acute* *Symmetric or slightly asymmetric* *Green* *High*
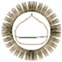	Bur: shape Bur: density of prickles	*Obloid* *Sparse*
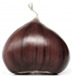	Nut: embryony Nut: degree of seed coat penetration into embryo Nut: shape Nut: area of pubescence on upper part Nut: area of hilum Nut: shape of border line of hilum and pericarp Nut: conspicuousness of hilum Nut: glossiness Nut: color of skin Nut: size	*Mono-embryonic* *Medium* *Circular* *Small* *Small* *Wavy* *Conspicuous* *Present* *Reddish brown* *Medium*
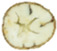	Seed coat: adherence to kernel Kernel: color of flesh	*Weak* *Yellow*
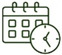	Time of leaf bud burst Time of male flowering Time of female flowering Time of maturity for consumption	*Medium* *Early* *Medium* *Early*

**Table 2:** Collection site cultivar and supplier, extraction methods and solvents, analytical tools used for chestnut shells’ characterization. TPP = Total Phenol Content; TFC = Total Flavonoid Content; TcTC = Total condensed Tannin Content; AA = Antioxidant Activity; EtOH = ethanol; MeOH = methanol; PET = Petroleum Ether; RT = Room Temperature; UAM = Ultrasound Assisted Maceration; SWE = Supercritical Water Extraction.

*Cultivar*	State	Extraction Method	Extraction Solvent	Analytical Tools	TPC	TFC	TcTC	AA	Refs.
–	Italy	Maceration (∅ 1–2 mm)	EtOH: H_2_O (different ratios *v*:*v*)	HPLC-UV-VIS	**X**	**x**			[22]
–	Spain	Acid hydrolysis (∅ 0.4 mm)	–	FTIR; TGA; MALDI-TOF-MS	**X**	**x**		**x**	[38]
Marrone di Roccadaspide	Italy	Maceration (RT)	MeOH, PET, CHCl_3_	LC-ESI/QTrap/MS^n^	**X**	**x**	**x**	**x**	[39]
–	Spain	Soxhlet	Several solvents	FT-IR; UV/Vis	**X**			**x**	[40]
–	Italy	SWE	H_2_O	LC-DAD/ESI-MS	**X**			**x**	[37]
–	Portugal	UAM	H_2_O	LC-UV-MS; ^1^H NMR	**X**			**x**	[41]
–	Portugal	Microwave	EtOH: H_2_O (different ratios, *v*:*v*)	LC-ESI/MS	**x**	**x**		**x**	[42]
–	Italy	Decoction	Boiling H_2_O (1 h)	UHPLC-MS/MS	**x**		**x**		[43]
Palomina	Italy	Ultra Turrax homogenizer; Decoction	Boiling H_2_O (30 min)	HPLC-UV	**x**			**x**	[44]
–	Spain	N.I.H	Alkaline solution or H_2_O	HPLC	**x**			**x**	[45]
–	Italy	Maceration (RT)	MeOH (60%—1:10, *w*:*v*); EtOH (60%—1:10, *w*:*v*); H_2_O (1:40, *w*:*v*)	HPLC-DAD	**x**		**x**	**x**	[46]
–	–	UAM (59 kHz)	EtOH: H_2_O (7:3, *v*:*v*)	HPLC-MS/MS	**x**	**x**		**x**	[47]
–	Bosnia	UAM (50 Hz and 125 W)	EtOH	–	**x**	**x**	**x**		[48]
Napoletana; Mercogliana Tempestiva; Bouche	Italy	Decoction	Boiling H_2_O (40 min)	HPLC-UV-DAD	**x**	**x**		**x**	[49]
–	Portugal	Maceration (RT)	EtOH	HPLC-DAD/ESI-MS	**x**		**x**	**x**	[50]
–	Italy	Maceration	Boiling H_2_O (1 h)		**x**	**x**	**x**	**x**	[16]
Judia		Maceration	EtOH	HPLC-UV	**x**	**x**	**x**		[51]
–	Italy	UAM (35 kHz)	MeOH	HPLC DAD/ESI-MS	**x**		**x**		[52]
–	Italy	–	MeOH (after defatting) EtOH:H_2_O (1:1, *v*:*v*)	UHPLC-UV/ESI-HRMS	**x**			**x**	[53]

**Table 3:** UHPLC-ESI-QqToF/MS and MS/MS data useful for the tentative identification of compounds in chestnut extracts (base peaks in MS/MS spectra are reported in bold; RT = retention time; RDB = ring and double bond value).

Peak	RT (Min)	Tentative Assignment	Formula	[M-H]^−^ Found (*m/z*)	Error (ppm)	RDB	MS/MS Fragment Ions (*m/z*) and Relative Intensity (%)
**1**	0.398	Hexahydroxydiphenoyl hexose	C_20_H_18_O_14_	481.0640	3.4	12	481.0638; **300.9986**; 275.0193; 257.0083
**2**	0.491	Gallic acid hexoside	C_13_H_16_O_10_	331.0663	−2.3	6	**169.0137**; 168.0073; 125.0245
**3**	0.674	Gallic acid	C_7_H_6_O_5_	169.0146	2.1	2	169.0146; **125.0248**
**4**	0.829	Galloyl-HHDP-hexose	C_27_H_22_O_18_	633.0739	0.9	17	**633.0761**; 300.9978; 275.0193
**5**	0.967	Crenatin	C_13_H1_8_O_9_	317.0886	2.5	5	317.0891; 155.0362; **137.0242**
**7**	1.187	Dihydroxybenzoic acid	C_7_H_6_O_4_	153.0200	4.4	5	**109.0299**; 108.0220;
**9**	1.579	diHHDP-hexose	C_34_H_24_O_22_	783.0709	2.9	23	**783.0707**; 481.0627; 300.9984; 275.0191
**11**	1.823	Castalagin/ vescalagin	C_34_H_30_O_31_	933.0673	−2.7	20	**933.0677**; 631.0593; 300.9977
**14**	4.838	Castacrenin	C_27_H_18_O_17_	613.0487	2.6	10	613.0511; **493.0076**; 300.9988
**15**	5.665	Digalloyl hexose	C_20_H_20_O_14_	483.0780	−0.1	11	483.0757; 313.0554; **211.0241**; 271.0462 169.0150; 125.0250;
**18**	8.019	Trigalloylhexose 1	C_27_H_24_O_18_	635.0890	0	16	**635.0947**; 483.0800; 465.0701; 423.0607; 313.0567; 169.0143
**19**	8.085	Trigalloylhexose 2	C_27_H_24_O_18_	635.0891	0.2	16	635.0932; 589.1926; 521.2044; **465.0707**; 359.1537; 313.0587; 169.0142
**26**	9.680	Ellagic acid	C_14_H_6_O_8_	300.9986	−1.3	12	**301.0000**; 283.9974; 185.0243
**31**	10.069	diHDDP-deoxyhexoside	C_34_H_22_O_20_	749.0643	1.5	24	**447.0579**; 300.9991; 299.9901
**41**	11.434	Methyl ellagic acid	C_15_H_8_O_8_	315.0145	−0.4	12.0	**299.9907**; 298.9814; 216.0057; 172.0169
**44**	12.245	Dimethyl ellagic acid deoxyhexoside	C_22_H_20_O_12_	475.0888	1.3	13.0	475.0912; 460.0681; 328.0228; **312.9991**; 297.9757
**46**	12.855	Dimethylellagic acid	C_16_H_10_O_8_	329.0304	0.3	12.0	329.0307; 314.0073; 298.9837; 285.0038; **270.9887**; 242.9934; 214.9982
**47**	14.272	Trimethylellagic acid eptuloside	C_24_H_24_O_14_	535.1109	2.9	13.0	343.0461; **328.0230**; 312.9991; 297.9749
**51**	15.135	Trimethyl ellagic acid	C_17_H_12_O_8_	343.0462	0.8	12.0	312.9988; **297.9755**; 285.0036; 269.9804;213.9901

**Table 4:** UHPLC-ESI-QqToF/MS and MS/MS data useful for the tentative identification of flavanol and proanthocyanin compounds in chestnut extracts (base peaks in MS/MS spectra are reported in bold; RT = retention time; RDB = ring and double bond value).

Peak	RT (Min)	Tentative Assignment	Formula	[M-H]^−^ Found (*m/z*)	Error (ppm)	RDB	MS/MS Fragment Ions (*m/z*) and Relative Intensity (%)
**6**	1.147	Prodelphinidin B-type I	C_30_H_26_O_14_	609.1258	1.3	18	609.1278; **441.0834**; 423.0723; 305.0664; 177.0193; 483.0942; 261.0756
**D**	1.308	Prodelphinidin B-type II	C_30_H_26_O_14_	609.1247	−2	18	609.1258; 441.0837; **423.0724**; 305.0667; 177.0194
**8**	1.503	Gallocatechin	C_15_H_14_O_7_	305.0665	−0.6	9	305.0650; 261.0726; 237.0735; 219.0661; 179.0337; 167.0342; 137.0240; **125.0243**; 111.0446
**10**	1.805	Procyanidin	C_30_H_26_O_13_	593.1289	−2.0	18	593.1312; 467.1045; 425.0900; **407.0795**; 303.0546; 289.0719; 177.0202
**E**	2.755	(epi)Catechin derivative I	C_30_H_26_O_14_	609.1244	−1	18	609.1249; 565.1344; 457.0764; 407.0758; 319.0452; 289.0706; **275.0553**; 231.0653; 165.0189
**12**	4.387	Catechin	C_15_H_14_O_6_	289.0719	0.5	9	289.0735; 271.0650; 245.0847; 221.0825; 205.0523; **203.0722**; 187.0414; 175.0404; 151.0418; 137.0251; 123.0457; 109.0312
**13**	4.564	Procyanidin B-type	C_30_H_26_O_12_	577.1349	−0.4	18	577.1399; 451.1277; 425.0900; **407.0788**; 289.0740; 245.0836; 125.0250
**F**	4.778	(epi)Catechin derivative II	C_30_H_26_O_14_	609.1245	−0.8	18	609.1257; 485.1241; 407.0771; **289.0696**; 231.0656; 206.0667; 102.0995
**16**	7.229	Procyanidin A-type	C_30_H_24_O_13_	591.1158	2.3	19	591.1226; **439.0687**; 421.0576; 285.0419; 177.0183
**21**	8.145	Procyanidin B-type *O*-gallate	C_37_H_30_O_16_	729.1475	1.9	23	**729.1506**; 577.1385; 451.1053; 425.0894; 407.0778; 289.0723; 245.0450; 125.0267
**22**	8.379	(epi)Catechin gallate	C_22_H_18_O_10_	441.0845	4.0	14	**441.0796**; 289.0731
**32**	10.073	Procyanidin B-type gallate	C_37_H_30_O_16_	729.1452	−1.2	23	729.1507; 577.1343; **407.0781**; 289.0715; 269.0462; 125.0245

**Table 5:** UHPLC-ESI-QqToF/MS and MS/MS data useful for the tentative identification of flavonoids in chestnut extracts (base peaks in MS/MS spectra are reported in bold; RT = retention time; RDB = ring and double bond value).

Peak	RT (Min)	Tentative Assignment	Formula	[M-H]^−^ Found (*m/z*)	Error (ppm)	RDB	MS/MS Fragment Ions (*m/z*) and Relative Intensity (%)
**23**	8.842	Dihydrokaempferol deoxyhexoside	C_21_H_22_O_11_	449.1097	1.7	11	449.1094; 287.0557; **269.0444**; 151.0035
**24**	9.395	Myricetin hexoside	C_21_H_20_O_13_	479.0827	−0.9	12	479.0871; 317.0336; **316.0234**; 287.0219
**25**	9.494	Dihydroquercetin galloyl hexoside (e.g., taxillusin)	C_28_H_26_O_16_	617.1152	0.6	5	**617.1215**; 491.0869; 465.1078; 313.0584; 303.0520; 194.9932
**27**	9.682	Myricetin deoxyhexoside	C_21_H_20_O_12_	463.0882	0	12	463.0910; 317.0315; **316.0238**; 287.0207; 316.0238; 271.0254
**28**	9.688	Quercetin hexoside gallate	C_21_H_28_O_21_	615.1050	−0.7	8.0	615.1046; **463.0907**; 301.0373; 300.0281
**29**	10.014	Phloretin hexoside	C_21_H_24_O_10_	435.1296	−0.2	10.0	345.0967; **315.0860**; 273.0772; 209.0455; 167.0348; 123.0450
**30**	10.033	Quercetin hexoside	C_21_H_20_O_12_	463.0892	2.2	12.0	463.0892; 301.0251; **300.0280**; 271.0242
**33**	10.290	Patuletin hexoside	C_22_H_22_O_13_	493.0986	−0.3	12	493.1081; 331.0468; **315.0141**; 287.0188; 271.0237; 151.0033
**34**	10.539	Dihydroquercetin	C_15_H_12_O_7_	303.0514	1.2	10.0	303.0498; 151.0409; **123.0448**
**35**	10.870	Quercetin deoxyhexoside	C_21_H_20_O_11_	447.0936	0.7	12.0	447.0964; **300.0291**; 271.0261; 151.0028
**36**	11.055	Isorhamnetin rutinoside	C_28_H_32_O_16_	623.1619	0.2	13.0	623.1675; **315.0527**; 314.0440; 300.0264
**37**	11.115	Isorhamnetin deoxyhexoside	C_22_H_22_O_12_	477.1036	−0.5	12.0	331.0480; **314.0456**; 299.0212; 271.0267
**38**	11.185	Naringenin gallate	C_22_H_16_O_9_	423.0723	0.3	15	423.0747; 299.0204; 271.0253; **258.0168**; 243.0303
**39**	11.331	Trihydroxy dimethoxyflavanol hexoside	C_23_H_24_O_13_	507.1146	0.4	12	507.1195; 345.0639; **344.0559**; 273.0418
**40**	11.653	Isorhamnetin pentoside	C_21_H_20_O_11_	447.0937	0.9	12	447.0936; 315.0527; **314.0440**; 300.0285; 285.0416; 271.0245; 243.0300
**42**	12.035	Isorhamnetin hexoside	C_22_H_22_O_11_	461.1093	0.8	12.0	461.1110; 315.0500; **314.0433**; 271.0240; 243.0284
**43**	12.315	Dihydroxy trimethoxyflavone deoxyhexoside	C_23_H_24_O_12_	491.1205	2.0	12.0	491.1244; 345.0637; **344.0558**; 329.0323; 315.0164; 301.0367; 273.0412
**45**	14.507	Isokaempferide	C_16_H_12_O_6_	299.0559	−0.7	11.0	**284.0322**; 256.0370; 255.0286; 227.0343; 151.0038
**48**	14.510	Methoxyquercetin	C_16_H_12_O_7_	315.0512	0.6	11	315.0530; **300.0257**; 283.0235; 271.0240; 255.0287; 151.0031
**49**	14.609	Dimethoxyquercetin	C_16_H_14_O_8_	345.0615	−0.3	11	345.0607; 330.0362; **315.0144**; 287.0186; 259.0240; 203.0388; 171.0452

**Table 6:** UHPLC-ESI-QqToF/MS and MS/MS data useful for the tentative identification of lipid compounds in chestnut extracts (base peaks in MS/MS spectra are reported in bold; RT = retention time; RDB = ring and double bond value).

Peak	RT (Min)	Tentative Assignment	Formula	[M-H]^−^ Found (*m/z*)	Error (ppm)	RDB	MS/MS Fragment Ions (*m/z*) and Relative Intensity (%)
**50**	14.665	Trihydroxyoctadecadienoic acid	C_18_H_32_O_5_	327.2180	0.9	3	327.2196;229.1437; 211.1335; **171.1026**
**52**	17.309	Chestnoside	C_36_H_54_O_11_	661.3606	1.9	10.0	**661.3650**; 499.3099; 419.2973
**53**	17.557	Bartogenic acid	C_30_H_46_O_7_	517.3182	2.2	8.0	**517.3209**; 455.3186; 437.3076
**54**	19.959	Dihydroxyursadiendioic acid (I)	C_30_H_44_O_6_	499.3075	2.0	9.0	**499.3075**; 455.3160; 437.3050; 419.2946
**55**	19.967	Hederagenin	C_29_H_44_O_5_	471.3116	2.1	8.0	471.3119; 453.3013; **409.3107**; 379.3000; 363.2712
**56**	20.369	Dihydroxyursadiendioic acid (II)	C_30_H_44_O_6_	499.3065	0.1	9.0	**499.3085**

## Data Availability

Data are contained within the article and Supplementary Materials.

## Supplementary Materials

The following supporting information can be downloaded at: https://www.mdpi.com/article/10.3390/molecules27248924/s1. Table S1. UPOV traits of *Castanea sativa* cv. Verdole.

## Sample Availability

Samples of the investigated extract and its fractions are available from the authors.
