# Supplementary material for: A Joint Approach of Morphological and UHPLC-HRMS Analyses to Throw Light on the Autochthonous ‘Verdole’ Chestnut for Nutraceutical Innovation of Its Waste
Source: Molecules. 2022 Dec 15;27(24):8924. doi: 10.3390/molecules27248924 (PMC9785621; doi:10.3390/molecules27248924)
Supplement: Supplementary file 1 [file molecules-27-08924-s001.zip › molecules-2100457-supplementary.pdf]

Supplementary materials

Table S1. UPOV traits of *Castanea sativa* cv Verdole

Species	Genetic resource	Accession
**Chestnut (*Castanea sativa* Mill.)**	**Verdole**	

Num				
	Image	Attributed	Variety of reference	**Attribute value**
1	Descriptor	Tree: vigor		
		Weak	Hong Mao Zao (C), Toyotamawase(B)	3 ☐
		Medium	Ibuki (B), Ishizuchi (B), Zhong Chi Li (C)	5 ☒
		Strong	Da Hong Pao (C), Ganne (B), Tsukuba (B)	7 ☐
	Image	Attributed	Variety of reference	**Attribute value**
2	Descriptor	Tree: growth habit		
	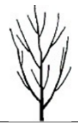	Upright	Akatyu (B), Arima (B), Bouche rouge (A), Song Jia Zao (C), Tsukuba (B)	1☒
	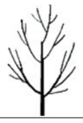	Semi-upright	Maraval (A), Otomune (B), Rihei (B), Yan Hong (C)	2 ☐
	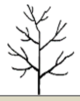	Spreading	Belle Epine (A), Ibuki (B), Zhong Chi Li (C	3 ☐
	Image	Attributed	Variety of reference	**Attribute value**
3	Descriptor	Current season's shoot: thickness		
		Thin	Arima (B), Ginrei (B), Marsol (A)	1 ☐
		Medium	Ginyose (B), Ishizuchi (B), Marron de Chevanceaux (A), Tanzawa (B)	3 ☐
		Thick	Belle Epine (A), Ibuki (B), Tsukuba (B)	5 ☒
	Image	Attributed	Variety of reference	**Attribute value**
4	Descriptor	Current season's shoot: length of internodes		
		Short	Ibuki (B), Marigoule (A), Yanshan Duan Zhi (C)	3 ☐
		Medium	JonGanne (B), Kui Li (C), Maraval (A), Shihou (B)	5 ☐
		Long	Jiu Yue Han (C), Marsol (A), Rihei (B)	7 ☒
	Image	Attributed	Variety of reference	**Attribute value**
5	Descriptor	Current season's shoot: arrangement of leaves		

	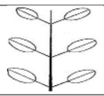	Opposite	Marsol (A)	**1 ☒**
	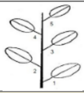	Alternate	Belle Epine (A)	**2 ☒**
	Image	Attributed	Variety of reference	**Attribute value**
**6**	Descriptor	Current season's shoot: the color of the upper side of the stem		
		Yellow-brown	Ganne (B), Ishizuchi (B), Okkwang (B), Shen Ci Da Ban Li (C)	**1 ☒**
		Brown	Ginyose (B), Tsukuba (B)	**2 ☐**
		Red-brown	Arima (B), Hong Guang You Li (C), Imakita (B), Tamatsukuri (B)	**3 ☐**
	Image	Attributed	Variety of reference	**Attribute value**
**7**	Descriptor	Current season's shoot: density of lenticels		
		Sparse	Marsol (A), Yan Kui (B)	**1 ☐**
		Medium	Da Ban Hong (C), Ginyose (B), Ibuki (B), Rousse de Nay (A), Tanzawa (B), Tsukuba (B)	**3 ☐**
		Dense	Bournette (A), Ginrei (B), Tamatsukuri (B), Taziriginyose (B), Yin Feng (C)	**5 ☒**
	Image	Attributed	Variety of reference	**Attribute value**
**8**	Descriptor	Shoot: number of female flowers		
		Few	Moriwase (B)	**1 ☐**
		Medium	Tanzawa (B), Tsukuba (B)	**3 ☐**
		Many	Arima (B), Ishizuchi (B)	**5 ☒**
	Image	Attributed	Variety of reference	**Attribute value**
**9**	Descriptor	Male flower: length of filament		
		Very short	Bouche rouge (A)	**1 ☐**
		Short	Marron d' Olargues (A)	**2 ☐**
		Medium	Marron de Redon (A)	**3 ☐**
		Long	Belle Epine (A)	**4 ☐**
		Very long		**5 ☒**
	Image	Attributed	Variety of reference	**Attribute value**
	Descriptor	Catkin: length		

**10**		Short	Belle Epine (A), Ganne (B), Ishizuchi (B), Jiu Jia Zhong (C), Toyotamawase (B)	**3 ☐**
		Medium	Akatyu (B), Da Di Qing (C), Ginyose (B), Izumo (B), Marron de Goujo unac (A)	**5 ☐**
		Long	Arima (B), Chu Shu Hong (C), Ibuki (B), Marron de Chevanceaux (A), Tanzawa (B), Tsukuba (B)	**7 ☒**
	Image	Attributed	Variety of reference	**Attribute value**
**11**	Descriptor	Young leaf: bronze coloration		
		Absent	Bouche rouge (A)	**1 ☒**
		Present	Belle Epine (A)	**9 ☐**
	Image	Attributed	Variety of reference	**Attribute value**
**12**	Descriptor	Leaf: size		
		Small	Maraval (A), Moriwase (B), Toyotamawase (B), Wu Hua Li (C)	**3 ☐**
		Medium	Bournette (A), Ginyose (B), Ibuki(B), Kui Li (C), Tanzawa (B)	**5 ☐**
		Large	Marsol (A), Qian Ci Da Ban Li (C), Riheiguri (B), Tsukuba (B)	**7 ☒**
	Image	Attributed	Variety of reference	**Attribute value**
**13**	Descriptor	Leaf: profile in cross-section		
		Straight	Belle Epine (A)	**1 ☐**
	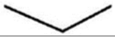	Slightly concave		**2 ☒**
	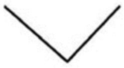	Strongly concave	Comballe (A)	**3 ☐**
	Image	Attributed	Variety of reference	**Attribute value**
**14**	Descriptor	Leaf: symmetry		
		Symmetric to slightly asymmetric	Marsol (A)	**1 ☐**
		Moderately asymmetric		**2 ☒**
		Strongly asymmetric	Bournette (A)	**3 ☐**
	Image	Attributed	Variety of reference	**Attribute value**
**15**	Descriptor:	Leaf: length/width ratio		
		Low	Marsol (A)	**3 ☐**
		Medium	Marron de Chevanceaux (A)	**5 ☐**
		High	Bournette (A)	**7 ☒**
	Image	Attributed	Variety of reference	**Attribute value**

**16**	Descriptor	Leaf: attitude in relation to shoot		
	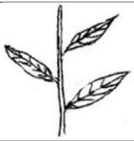	Upwards	Bouche rouge (A)	**1 ☒**
	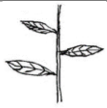	Outwards	Belle Epine (A)	**2 ☐**
	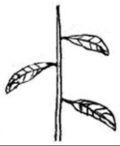	Downwards	Marron de Chevanceaux (A)	**3 ☐**
**17**	Image	Attributed	Variety of reference	**Attribute value**
	Descriptor:	Leaf blade: intensity of green color of upper side		
		Light	Belle Epine (A),<br>Da Di Qing (C)	**1 ☐**
		Medium	Er Xin Zao (C),<br>Ganne (B), Ginyose (B),<br>Rousse de Nay (A),<br>Tsukuba (B)	**3 ☒**
		Dark	Bouche rouge (A),<br>Dabufen Pinzho ng (C)	**5 ☐**
**18**	Image	Attributed	Variety of reference	**Attribute value**
	Descriptor	Leaf: color of lower side		
		Whitish	Banseki (B), Marsol (A)	**1 ☐**
		Light green	Bouche rouge (A),<br>Ginyose (B)	**2 ☒**
**19**	Image	Attributed	Variety of reference	**Attribute value**
	Descriptor	Leaf: shape		
	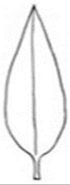	Lanceolate	Jiu Yue Han (C)	**1 ☐**
	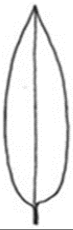	Narrow elliptic	Dae han (B), Ganne (B),<br>Ginyose (B), Mipung (B),<br>Qian Ci Da Ban Li (C),<br>Tsukuba (B)	**2 ☒**
	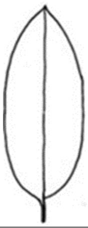	Broad elliptic	Zhong Chi Li (C)	**3 ☐**
**20**	Image	Attributed	Variety of reference	**Attribute value**
	Descriptor	Leaf: shape of apex		
		Narrow acuminate	Ishizuchi (B),<br>Qian Ci Da Ban Li (C),<br>Tanzawa (B),	**1 ☐**

	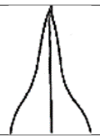		Tsukuba (B)	
	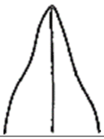	Broad acuminate	Ginyose (B), Ibuki (B), Jian Ding You Li (C)	2 ☒
	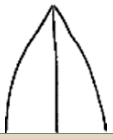	Acute	Ginrei (B), Imakita (B)	3 ☐
21	Image	Attributed	Variety of reference	**Attribute value**
	Descriptor	Leaf: the shape of base		
	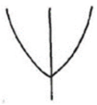	Acute	Bournette (A), Ginyose (B), Ibuki (B), Jiu Yue Han (C), Tanzawa (B)	1 ☐
	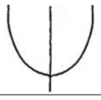	Obtuse	Qian Ci Da Ban Li (C), Verdale (A)	2 ☐
	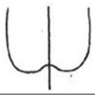	Cordate	Comballe (A), Hui Huang You Li (C)	3 ☒
22	Image	Attributed	Variety of reference	**Attribute value**
	Descriptor	Leaf: shape of margin		
	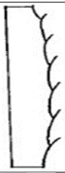	Needle shape	Ibuki (B), Ishizuchi (B), Tanzawa (B)	1 ☐
	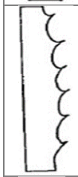	Acute	Akatyu (B), Izumo (B)	2 ☒
	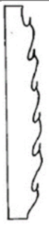	Flare shape	Marsol (A)	3 ☐
23	Image	Attributed	Variety of reference	**Attribute value**
	Descriptor	Leaf: symmetry of base		
		Symmetric or slightly asymmetric	Belle Epine (A)	1 ☒
		Moderately asymmetric		2 ☐
		Strongly asymmetric	Marsol (A)	3 ☐
24	Image	Attributed	Variety of reference	**Attribute value**
	Descriptor	Leaf: color of petiole		
		Yellow	Marsol (A)	1 ☐

		Green	Belle Epine (A)	2 ☒
25	Image	Attributed	Variety of reference	**Attribute value**
	Descriptor	Leaf: ratio length of leaf blade/length of petiole		
		Low	Arima (B), Maraval (A), Riheiguri (B), Tsukuba (B)	3 ☐
		Medium	Ginyose (B), Ishizuchi (B), Marsol (A), Tanzawa (B)	5 ☐
		High	Ganne (B), Ibuki (B), Toyotamawase (B), Verdale (A)	7 ☒
26	Image	Attributed	Variety of reference	**Attribute value**
	Descriptor	Bur: shape		
	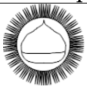 Front view	Globose	Ganne (B), Ibuki (B), Jiao Ci (C)	1 ☐
	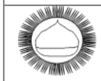 Front view 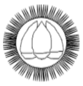 Lateral view	Obloid	Arima (B), Ishizuchi (B), Jiu Jia Zhong (C), Tanzawa (B), Tsukuba (B)	2 ☒
	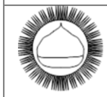 Front view 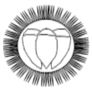 Lateral view	Transverse cylindric	Ginyose (B), Imakita (B)	3 ☐
27	Image	Attributed	Variety of reference	**Attribute value**
	Descriptor	Bur: density of prickles		
		Sparse	Duan Ci You Li (C), Tanzawa (B), Tsukuba (B)	1 ☒
		Medium	Cha Wan Li (C), Moriwase (B)	3 ☐
		Dense	Ginyose (B), Ishizuchi (B), Shen Ci Da Ban Li (C)	5 ☐
28	Image	Attributed	Variety of reference	**Attribute value**
	Descriptor	Nut: embryony		
	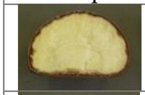	Mono-embryonic	Belle Epine (A)	1 ☒
	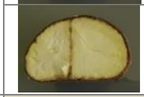	Poly-embryonic	Laguepie (A)	2 ☐
29	Image	Attributed	Variety of reference	**Attribute value**
	Descriptor	Poly-embryonic varieties only: Nut: coherence of embryos		
		Weak	Maraval (A)	3 ☐

		Medium	Precoce Migoule (A)	5 ☐
		Strong	Laguepie (A)	7 ☐
**30**	Image	Attributed	Variety of reference	**Attribute value**
	Descriptor	Nut: degree of penetration of seed coat into embryo		
		Absent or very weak	Marigoule (A)	1 ☐
		Weak	Maraval (A)	3 ☐
		Medium	Bournette (A)	5 ☒
		Strong	Laguepie (A)	7 ☐
**31**	Image	Attributed	Variety of reference	**Attribute value**
	Descriptor	Nut: shape		
	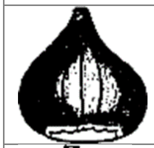	Broad ovate	Marsol (A)	1 ☐
	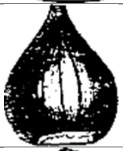	Medium ovate	Jian Ding You Li (C), Marki (A)	2 ☐
	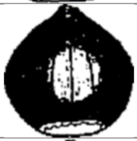	Circular	Arima (B), Da Hong Pao (C), Ishizuchi (B), Marron de Chevanceaux (A)	3 ☒
	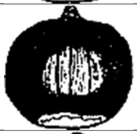	Medium oblate	Laguepie (A)	4 ☐
	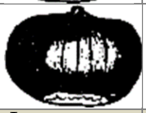	Broad oblate	Izumo (B), Marigoule (A), Qian Ci Da Ban Li (C), Riheiguri (B)	5 ☐
**32**	Image	Attributed	Variety of reference	**Attribute value**
	Descriptor	Nut: area of pubescence on upper part		
	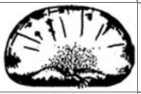	Small	Ginyose (B), Tamatsukuri (B), Tsukuba (B), You Li (C)	1 ☒
	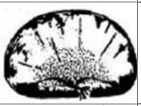	Medium	Ibuki (B), Ishizuchi (B), Tanzawa (B)	3 ☐
	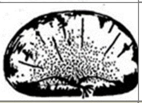	Large	Ganne (B), Riheiguri (B), Yang Mao Li (C)	5 ☐
**33**	Image	Attributed	Variety of reference	**Attribute value**
	Descriptor	Nut: area of hilum		
	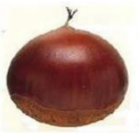	Small	Comballe (A), Da Ban Hong (C), Ishizuchi (B), Riheiguri (B), Toyotamawase (B)	3 ☐
	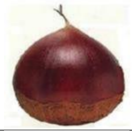	Medium	Ibuki (B), Marron d'Olargues (A), Tanzawa (B), Tsukuba (B), Yanshan Zao Feng (C)	5 ☒
	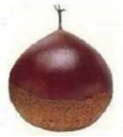	Large	Arima (B), Da Di Qing (C), Ganne (B), Ginrei (B), Marigoule (A)	7 ☐
**34**	Image	Attributed	Variety of reference	**Attribute value**
	Descriptor	Nut: shape of border line of hilum and pericarp		

	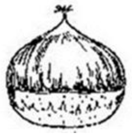	Straight	Arima (B), Cui Jia Bao Zi 2399 (C), Imakita (B)	**1** ☐
	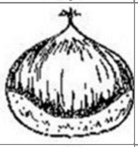	Curved	Hong Li (C), Ibuki (B), Tanzawa (B), Tsukuba (B)	**2** ☐
	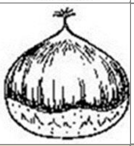	Wavy	Ganne (B), Otomune (B), Riheiguri (B), Xinyang Da Ban Li (C)	**3** ☒
**35**	Image	Attributed	Variety of reference	**Attribute value**
	Descriptor	Nut: conspicuousness of hilum		
		Inconspicuous	Rousse de Nay (A)	**1** ☐
		Conspicuous	Marigoule (A)	**2** ☒
**36**	Image	Attributed	Variety of reference	**Attribute value**
	Descriptor	Nut: glossiness		
		Absent	Marigoule (A)	**1** ☐
		Present	Belle Epine (A)	**9** ☒
**37**	Image	Attributed	Variety of reference	**Attribute value**
	Descriptor	Nut: color of skin		
		Light brown	Comballe (A), Hangawii (B), Hong Guang (C), Otomune (B), Tanzawa (B)	**1** ☐
		Medium brown	Arima (B), Belle Epine (A), Mipung (B), Okkwang (B), Taziriginyose (B), Zhong Chi Li (C)	**2** ☐
		Dark brown	Akatyu (B), Ishizuchi (B), Jiao Zha (C), Tsukuba (B)	**3** ☐
		Reddish brown	Daekwang (B), Ganne (B), Ginyose (B), Ibuki (B), Liu Yue Pu (C), Marron de Var (A)	**4** ☒
		Blackish brown	Marigoule (A), Riheiguri (B), WuKe Li (C)	**5** ☐
**38**	Image	Attributed	Variety of reference	**Attribute value**
	Descriptor	Nut: size		
		Small	Hangan Tie Dan Li (C), Imakita (B), Roussette de Montpazier (A), Toyotamawase (B)	**3** ☐
		Medium	Arima (B), Ibuki (B), Laguepie (A), Tanzawa (B), Yan Hong (C)	**5** ☒
		Large	Ganne (B), Ginyose (B), Marigoule (A), Tsukuba (B), Xinyang Da Ban Li (C)	**7** ☐
**39**	Image	Attributed	Variety of reference	**Attribute value**
	Descriptor	Seed coat: adherence to kernel		
		Weak	Marigoule (A), Riheiguri (B)	**3** ☒
		Medium	Akatyu (B), Ishizuchi (B), Tanzawa (B)	**5** ☐
		Strong	Ginyose (B), Ibuki (B), Laguepie (A), Tsukuba (B)	**7** ☐
**40**	Image	Attributed	Variety of reference	**Attribute value**
	Descriptor	Kernel: color of flesh		
		White	Akatyu (B), Ginrei (B), Hubei You Li (C),	**1** ☐

			Imakita (B), Marigoule (A)	
		Whitish yellow	Arima (B), Belle Epine (A), Ginyose (B), Hangawii (B), Ishizuchi (B), Okkwang (B), Yu Luo Hong(C)	**2** ☐
		Yellow	Ibuki (B), Mipung (B), Riheiguri (B), Tanzawa (B), Tsukuba (B), Zhong Chi Ban Li (C)	**3** ☒
**41**	Image	Attributed	Variety of reference	**Attribute value**
	Descriptor	Mono-embryonic varieties only: Kernel: inner cavity		
		Absent	Belle Epine (A)	**1** ☐
		Present	Bouche rouge (A)	**9** ☐
**42**	Image	Attributed	Variety of reference	**Attribute value**
	Descriptor	Time of leaf bud burst		
		Very early	Maraval (A), Shen Ci Da Ban Li (C)	**1** ☐
		Early	Ginyose (B), Precoce de Vans (A), Toyotamawase (B), Zao Li Zi (C)	**3** ☐
		Medium	Doree de Lyon (A), Er Hung Zao (C), Ganne (B), Tanzawa (B), Tsukuba (B)	**5** ☒
		Late	Arima (B), Ishizuchi (B), Marron Dauphine (A), Riheiguri (B), Yan Chang (C)	**7** ☐
		Very late	Banseki (B), Marron Comballe (A), Yin Feng (C)	**9** ☐
**43**	Image	Attributed	Variety of reference	**Attribute value**
	Descriptor	Time of male flowering		
		Very early	Moriwase (B), Shandong Lai Xi Da You Li (C), Soulage Premiere (A)	**1** ☐
		Early	Akatyu (B), Marigoule (A), Qing Mao Zao (C), Tamatsukuri (B), Toyotamawase (B)	**3** ☒
		Medium	Chu Shu Hong (C), Ginyose (B), Ibuki (B), Marron de Chevanceaux (A), Tanzawa (B)	**5** ☐
		Late	Belle Epine (A), Ganne (B), Ishizuchi (B), Jiu Jia Zhong (C), Tsukuba (B)	**7** ☐
		Very late	Banseki (B), Jiu Hua 2 (C), Marron de Goujo unac (A)	**9** ☐
**44**	Image	Attributed	Variety of reference	**Attribute value**
	Descriptor	Time of female flowering		
		Very early	Chu Shu Hong (C), Moriwase (B), Soulage Premiere (A)	**1** ☐
		Early	Akatyu (B), Jiu Jia Zhong (C), Marigoule (A), Tamatsukuri (B)	**3** ☐
		Medium	Arima (B), Bouche rouge (A), Hua Guang (C), Ibuki (B)	**5** ☒
		Late	Belle Epine (A), Ishizuchi (B), Qing Mao Ruan Ci (C)	**7** ☐
		Very late	Banseki (B), Verdale (A)	**9** ☐
**45**	Image	Attributed	Variety of reference	**Attribute value**
	Descriptor	Time of maturity for consumption		
		Very early	Bouche de Betizac (A),	**1** ☐

			Eli1 (C), Moriwase (B),<br>Toyotamawase (B)	
		Early	Izumo (B),<br>Precoce Migoule (A),<br>Song Jia Zao (C),<br>Tamatsukuri (B),<br>Tanzawa (B)	**3** ☒
		Medium	Arima (B),<br>Hua Guang (C),<br>Marigoule (A),<br>Tsukuba (B)	**5** ☐
		Late	Bouche rouge (A),<br>Ganne (B), Ishizuchi (B),<br>Qing Mao Ruan Ci (C)	**7** ☐
		Very late	Banseki (B), Verdale (A)	9 ☐
